# 2038 Effects of bilateral frontal transcranial direct current stimulation (tDCS) on the working memory network: An fMRI-tDCS study in healthy older adults

**DOI:** 10.1017/cts.2018.70

**Published:** 2018-11-21

**Authors:** Nicole R. Nissim, Andrew O’Shea, Lindsey Richards, Rachel Telles, Eric Porges, Ronald Cohen, Adam J. Woods

**Affiliations:** 1 Center for Cognitive Aging and Memory, McKnight Brain Institute, Department of Clinical and Health Psychology, University of Florida, Gainesville, FL, USA; 2 Department of Neuroscience, University of Florida, Gainesville, FL, USA

## Abstract

OBJECTIVES/SPECIFIC AIMS: The study aimed to determine the effects of bilateral frontal active transcranial direct current stimulation (tDCS) at 2 mA for 12 minute Versus sham stimulation on functional connectivity of the working memory network during an fMRI N-Back task. METHODS/STUDY POPULATION: Stimulation was delivered over bilateral frontal dorsolateral prefrontal cortex via and MRI-compatible tDCS device during an fMRI working memory task in healthy older adults in a within-subject design. RESULTS/ANTICIPATED RESULTS: Active stimulation compared with sham resulted in significant increases in functional connectivity in working memory related brain regions during the N-Back task. DISCUSSION/SIGNIFICANCE OF IMPACT: Older adults typically have reduced functional connectivity compared with young adults. Our findings demonstrate that a single session of tDCS can increase functional connectivity of the working memory network in older adults. Based on this mechanism of effect, tDCS may serve as an adjunctive method for interventions aiming to enhance cognitive processes in older adults.

